# Melatonin MT_1_ and MT_2_ Receptors Exhibit Distinct Effects in the Modulation of Body Temperature across the Light/Dark Cycle

**DOI:** 10.3390/ijms20102452

**Published:** 2019-05-17

**Authors:** Martha López-Canul, Seung Hyun Min, Luca Posa, Danilo De Gregorio, Annalida Bedini, Gilberto Spadoni, Gabriella Gobbi, Stefano Comai

**Affiliations:** 1 Neurobiological Psychiatry Unit, Department of Psychiatry, McGill University Health Center (MUHC), McGill University, Montreal, QC H3A1A1, Canada; martha.lopezcanul@mcgill.ca (M.L.-C.); seung.min@mail.mcgill.ca (S.H.M.); luca.posa@mail.mcgill.ca (L.P.); danilo.degregorio@mail.mcgill.ca (D.D.G.); comai.stefano@hsr.it (S.C.); 2 Alan Edwards Centre for Research on Pain, McGill University, Montreal, QC H3A 0G1, Canada; 3 Department of Biomolecular Sciences, University Carlo Bo, 61029 Urbino, Italy; annalida.bedini@uniurb.it (A.B.); gilberto.spadoni@uniurb.it (G.S.); 4 Neuropsychopharmacology Unit, San Raffaele Scientific Institute and Vita-Salute University, 20132 Milan, Italy

**Keywords:** melatonin, MT_1_ receptors, MT_2_ receptors, MT_3_ receptors, body temperature, light/dark cycle

## Abstract

Melatonin (MLT) is a neurohormone that regulates many physiological functions including sleep, pain, thermoregulation, and circadian rhythms. MLT acts mainly through two G-protein-coupled receptors named MT_1_ and MT_2_, but also through an MLT type-3 receptor (MT_3_). However, the role of MLT receptor subtypes in thermoregulation is still unknown. We have thus investigated the effects of selective and non-selective MLT receptor agonists/antagonists on body temperature (T_b_) in rats across the 12/12-h light–dark cycle. Rectal temperature was measured every 15 min from 4:00 a.m. to 9:30 a.m. and from 4:00 p.m. to 9:30 p.m., following subcutaneous injection of each compound at either 5:00 a.m. or 5:00 p.m. MLT (40 mg/kg) had no effect when injected at 5 a.m., whereas it decreased T_b_ during the light phase only when injected at 5:00 p.m. This effect was blocked by the selective MT_2_ receptor antagonist 4P-PDOT and the non-selective MT_1_/MT_2_ receptor antagonist, luzindole, but not by the α_1_/MT_3_ receptors antagonist prazosin. However, unlike MLT, neither the selective MT_1_ receptor partial agonist UCM871 (14 mg/kg) nor the selective MT_2_ partial agonist UCM924 (40 mg/kg) altered T_b_ during the light phase. In contrast, UCM871 injected at 5:00 p.m. increased T_b_ at the beginning of the dark phase, whereas UCM924 injected at 5:00 a.m. decreased T_b_ at the end of the dark phase. These effects were blocked by luzindole and 4P-PDOT, respectively. The MT_3_ receptor agonist GR135531 (10 mg/kg) did not affect T_b_. These data suggest that the simultaneous activation of both MT_1_ and MT_2_ receptors is necessary to regulate T_b_ during the light phase, whereas in a complex but yet unknown manner, they regulate T_b_ differently during the dark phase. Overall, MT_1_ and MT_2_ receptors display complementary but also distinct roles in modulating circadian fluctuations of T_b_.

## 1. Introduction

The maintenance of body temperature (T_b_) in mammalians is critical for survival and internal homeostasis. The main brain structure involved in controlling T_b_ is the hypothalamus, which receives inputs from the thermoreceptors located in both the brain and the periphery. Depending on these inputs, homeostatic changes are subsequently induced, causing sweating or shivering [1]. In particular, the preoptic area (POA) and dorsomedial hypothalamus (DMH) are critical hypothalamic areas for thermoregulation. In these areas, based on the firing rate responses to changes in local brain temperature, electrophysiological recordings have shown three different neuronal populations: warm-sensitive neurons (~30%), cold-sensitive neurons (~6%), and insensitive neurons (~60%), [2,3]. Activation of the thermally-responsive GABAergic and glutamatergic neurons in the ventral part of the lateral preoptic nucleus (vLPO) and the dorsal part of the dorsomedial hypothalamus (DMD), respectively, decreases temperature, physical activity, and metabolic rate. On the contrary, GABAergic neurons in the DMD promote the increase of T_b_, energy expenditure, and physical activity [4].

Recently, it has also been found that T_b_ can be internally regulated in a circadian manner by endogenous signals from other parts of the hypothalamus [4,5], specifically in the suprachiasmatic nucleus (SCN) [1,6,7]. The SCN regulates the circadian rhythmicity of several physiological responses [1], including the oscillatory decrease of the thermoregulatory threshold of heat production during day time and heat loss during night time in diurnal species [1,8,9]. However, the mechanisms underlying this circadian modulation of T_b_ remain unclear. Interestingly, the hypothalamus, including the SCN, DMH, and POA, are rich in melatonin (MLT) MT_1_ and MT_2_ receptors [7,10,11,12,13], and the activation of these MLT receptors modulates numerous physiological effects including the control of T_b_ [14].

MLT is produced in the pineal gland, mostly during the dark phase in both diurnal and nocturnal species [12,15]. The circadian production and release of MLT are controlled by the SCN [16]. Most of the physiological effects of MLT result from the activation of two high-affinity G-protein coupled receptors (GPCRs), MT_1_ (pK_i_ = 10.09) and MT_2_ (pK_i_ = 9.42), both of which are widely expressed in the mammalian brain [7,10,17]. The specific localization of the two MLT receptor subtypes in different regions of the brain and/or neuronal populations [11,18,19,20] partially explains the selective and differential functional activity of the two MLT receptor subtypes, such as in sleep [18,21,22,23], anxiety [24], pain [25,26], circadian rhythms [27], and depression [28]. In addition to these high-affinity MLT receptors, another low-affinity MLT binding site, termed MT_3_ (pKi = 6.0), has been reported [29,30]. Given its both hydrophilic and lipophilic nature, MLT can easily pass through the cell membrane and bind nuclear receptors, including retinoic acid receptor-related orphan receptors (RORs) [31].

It is known that MLT decreases T_b_ during the night in diurnal species [1]. Clinical studies have shown that exogenous administration of MLT suppresses the physiological increase in T_b_ observed during daytime [32,33] and has hypothermic properties at the dose of 5 mg/kg [34,35,36,37,38]. Similar results have been demonstrated in preclinical studies in diurnal animals in which administration of MLT acted as a hypothermic agent in the active/light phase in fat sand rats and Marshall broiler chickens [39,40]. However, the neurobiological mechanism through which MLT exerts this hypothermic effect, as well as the selective contribution of the three MLT receptor subtypes, are yet to be investigated.

Therefore, we investigated modifications in T_b_ produced by selectively activating the three MLT receptor subtypes across the light–dark cycle in rats. To achieve this aim, we tested the effects of the selective MT_2_ receptor partial agonist N-{2-[(3-bromophenyl)-(4-fluorophenyl)amino] ethyl}acetamide (UCM924) (pK_iMT1_ = 6.76; pK_iMT2_ = 9.27) [41], the selective MT_1_ receptor partial agonist N-(2-{Methyl-[3-(4-phenylbutoxy)phenyl]amino}ethyl) acetamide (UCM871) (pK_iMT1_ = 8.93; pK_iMT2_ = 7.04) [42], and the MT_3_ receptor agonist 5-Methoxycarbonylamino-N-acetyltryptamine (GR135531) (pK_iMT3_ = 29.5) on T_b_. The effects of UCM924, UCM871, and GR135531 were compared to those of MLT. In addition, selective and non-selective MLT receptor antagonists were also tested together with MLT and the other MLT receptor agonists/partial agonists to further dissect the MLT receptor subtypes involved in their thermoregulatory effects.

## 2. Results

**Figure 1:**
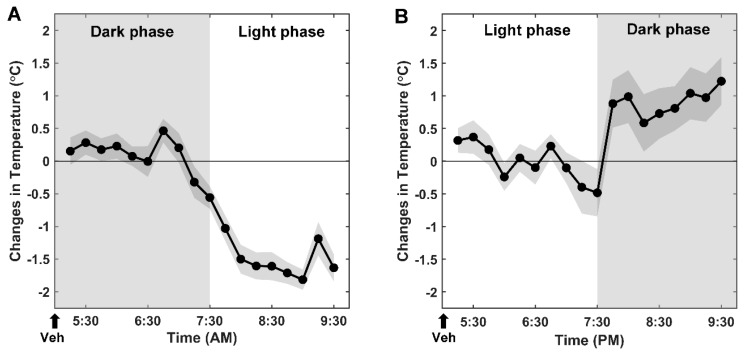
Body temperature (T_b_) changes during the light–dark cycle in rats exposed to a 12/12-h light–dark cycle. (**A**) T_b_ decreases during the transition from the dark phase to the light phase. (**B**) T_b_ increases during the transition from the light phase to the dark phase. Lights on at 7:30 a.m. and off at 7:30 p.m. Data represent mean value ± SEM. Veh: s.c. injection of vehicle.

In physiological conditions, as already known [1,9], there are changes in T_b_ between the light and the dark phase; in particular, T_b_ oscillations mostly occur during the phase shift (Figure 1). During the shift from the dark to the light phase, the T_b_ drops from an average of 38.45 to 37.5 °C after the light turns on (Figure 1A). The opposite occurs during the shift from the light to the dark phase when the light is turned off (Figure 1B).

### 2.1. Effects of MLT Injected at the End of the Dark and of the Light Phases on T_b_

**Figure 2:**
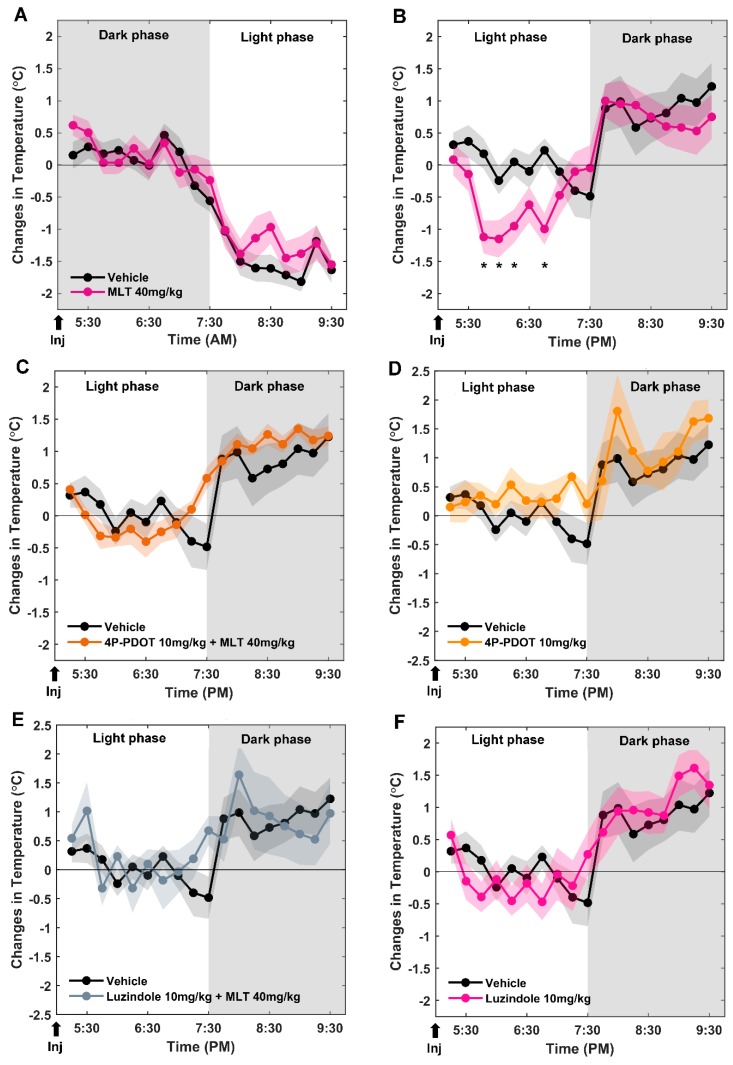
Changes in T_b_ after MLT administration (40 mg/kg) during the light and the dark phase. (**A**) MLT does not produce changes in T_b_ when administrated at 5:00 a.m. (**B**) MLT administrated during the dark phase (5:00 p.m.) decreases the T_b_ immediately after the administration compared with vehicle treated rats. (**C**) 4P-PDOT (10 mg/kg) pre-treatment blocks the effect of MLT on T_b_ during the light phase. (**D**) 4P-PDOT (10 mg/kg) injected during the light phase does not affect T_b_. (**E**) Pre-treatment with luzindole (10 mg/kg) blocks the effect of MLT on T_b_ during the light phase. (**F**) luzindole (10 mg/kg) injected during the light phase does not affect T_b_. Data are expressed as mean ± SEM (graded shades). Lights on at 7:30 a.m. and off at 7:30 p.m. * *p* < 0.05 vs. vehicle; two-way ANOVA for repeated measures followed by Bonferroni *post hoc* test. Inj: s.c. injection of either vehicle, MLT, MLT + 4P-PDOT, 4P-PDOT, MLT + luzindole, or luzindole.

As indicated in Figure 2A, the injection of MLT (40 mg/kg) at the end of the dark phase (5:00 a.m.) did not affect T_b_ during the end of the dark phase, the dark–light transition or the beginning of the light phase (two-way repeated measures ANOVA; interaction: F_17,323_ = 0.85, *p* = 0.635; treatment: F_1,323_ = 0.867, *p* = 0.363; time of the day: F_17,323_ = 31.835, *p* < 0.001).

In contrast, when MLT (40 mg/kg) was injected at the end of the light phase (5:00 p.m.), it induced a significant decrease (*p* < 0.05) in T_b_ from 5:45 p.m. to 6:45 p.m. that was close to the transition from the light to the dark phase (Figure 2B; interaction: F_17,408_ = 1.908, *p* = 0.016; treatment: F_1,408_ = 1.996, *p* = 0.171; time of the day: F_17,408_ = 10.658, *p* < 0.001). Importantly, we observed no further effects of MLT on T_b_ after the light–dark transition or during the beginning of the dark phase. The selective MT_2_ receptor antagonist 4P-PDOT at a dose not affecting T_b_ (Figure 2D; interaction: F_17,340_ = 0.62, *p* = 0.876; treatment: F_1,340_ = 2.07, *p* = 0.166; time of the day: F_17,340_ = 4.86, *p* < 0.001) blocked the effects of MLT (Figure 2C; interaction: F_17,391_ = 1.448, *p* = 0.111; treatment: F_1,391_ = 0.22, *p* = 0.643; time of the day: F_17,391_ = 11.486, *p* < 0.001). Similarly, the pre-treatment with the selective MT_1_/MT_2_ receptor antagonist luzindole at the dose not affecting T_b_ (Figure 2F; interaction: F_17,408_ = 1.144, *p* = 0.309; treatment: F_1,408_ = 0.012, *p* = 0.912; time of the day: F_17,408_ = 9.289, *p* < 0.001) also blocked the effects of MLT (Figure 2E; interaction: F_17,289_ = 0.989, *p* = 0.47; treatment: F_1,289_ = 0.11, *p* = 0.745; time of the day: F_17,289_ = 3.745, *p* < 0.001).

### 2.2. Effects of the Selective MT_2_ Partial Agonist UCM924 Injected at the End of the Dark and of the Light Phases on T_b_

**Figure 3:**
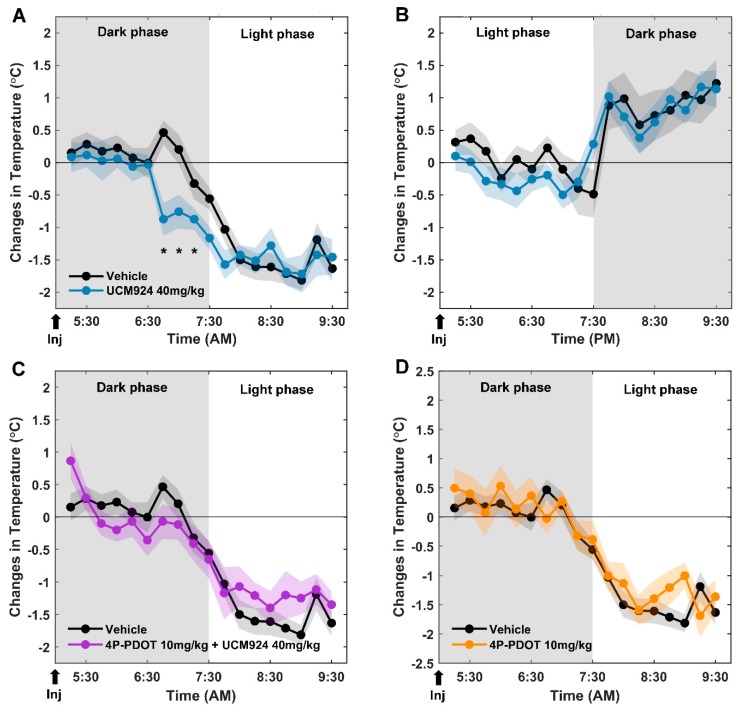
Changes in T_b_ after UCM924 (40 mg/kg, s.c.) treatment during the light and the dark phase. (**A**) UCM924 (40 mg/kg) administered at 5:00 a.m. (light phase) decreases T_b_ prior the light–dark shift compared with vehicle. (**B**) UCM924 (40 mg/kg) administered at 5:00 p.m. does not produce any change in the T_b_ compared with vehicle. (**C**) 4P-PDOT pre-treatment blocks the effect of UCM924 on T_b_ during the dark phase. (**D**) 4P-PDOT (10 mg/kg) injected during the dark phase does not affect T_b_. Data are expressed as mean ± SEM (graded shades). Lights on at 7:30 a.m. and off at 7:30 p.m. * *p* < 0.05 versus vehicle; two-way ANOVA for repeated measures followed by Bonferroni *post hoc* test. Inj: s.c. injection of either vehicle, UCM924, UCM924 + 4P-PDOT, or 4P-PDOT.

As indicated in Figure 3A, the injection of UCM924 (40 mg/kg) at the end of the dark phase (5:00 a.m.) induced a significant decrease (*p* < 0.05) in T_b_ immediately before the dark–light transition (from 6:45 a.m. to 7:30 a.m.), and did not affect T_b_ during the dark–light transition and at the beginning of the light phase (two-way repeated measures ANOVA; interaction: F_17,289_ = 0.2.406, *p* = 0.002; treatment: F_1,289_ = 2.286, *p* = 0.149; time of the day: F_17,289_ = 30.597, *p* < 0.001).

In contrast, when UCM924 (40 mg/kg) was injected during the light phase (5:00 p.m.), it did not affect T_b_ during the end of the light phase, the dark–light transition or the beginning of the dark phase (Figure 3B; two-way repeated measures ANOVA; interaction: F_17,425_ = 0.785, *p* = 0.711; treatment: F_1,425_ = 0.311, *p* = 0.582; time of the day: F_17,425_ = 9.618, *p* < 0.001).

The effects of UCM924 on T_b_ when injected during the dark phase were mediated by MT_2_ receptors since the pre-treatment with the selective MT_2_ receptor antagonist 4P-PDOT at a dose not affecting T_b_ (Figure 3D; interaction: F_17,272_ = 0.875, *p* = 0.605; treatment: F_1,272_ = 1.223, *p* = 0.285; time of the day: F_17,272_ = 22.848, *p* < 0.001) blocked the effects of UCM924 (Figure 3C; interaction: F_17,272_ = 1.922, *p* = 0.016; treatment: F_1,272_ = 0.0.35, *p* = 0.821; time of the day: F_17,272_ = 30.468, *p* < 0.001).

### 2.3. Effects of the Selective MT_1_ Partial Agonist UCM871 Injected at the End of the Dark and of the Light Phases on T_b_

**Figure 4:**
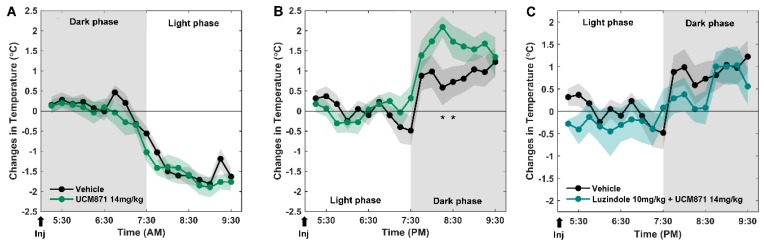
Changes in T_b_ after UCM871 (14 mg/kg, s.c.) treatment during the light and the dark phase. (**A**) UCM871 administered at 5:00 a.m. (dark phase) does not produce any change in the T_b_ compared with vehicle. (**B**) UCM871 administered at 5:00 p.m. (light phase) increases T_b_ after the light–dark transition compared with vehicle. (**C**) Luzindole pre-treatment blocks the effect of UCM871 on T_b_. Data are expressed as mean ± SEM (graded shades). Lights on at 7:30 a.m. and off at 7:30 p.m. * *p* < 0.05 versus vehicle; two-way ANOVA for repeated measures followed by Bonferroni *post hoc* test. Inj: s.c. injection of either vehicle, UCM871, or UCM871 + luzindole.

As indicated in Figure 4A, the injection of UCM871 (14 mg/kg) at the end of the dark phase (5:00 a.m.) did not affect T_b_ during the end of dark phase, the dark–light transition or the beginning of light phase (two-way repeated measures ANOVA; interaction: F_17,340_ = 0.842, *p* = 0.644; treatment: F_1,340_ = 0.538, *p* = 0.472; time of the day: F_17,340_ = 44.622, *p* < 0.001).

In contrast, when UCM871 (14 mg/kg) was injected during the light phase (5:00 p.m.), it induced a significant increase (*p* < 0.05) in T_b_ from 8:15 p.m. to 8:45 p.m. (dark phase) (Figure 4B; interaction: F_17,340_ = 1.634, *p* = 0.05; treatment: F_1,340_ = 2.045, *p* = 0.168; time of the day: F_17,340_ = 10.42, *p* < 0.001). The effects of UCM871 on T_b_ when injected during the light phase were blocked by the pre-treatment with the MT_1_/MT_2_ receptor antagonist luzindole (Figure 4C; interaction: F_17,340_ = 0.67, *p* = 0.83; treatment: F_1,340_ = 1.22, *p* = 0.28; time of the day: F_17,340_ = 4.88, *p* < 0.001) at a dose not affecting T_b_ (see Figure 2F; interaction: F_17,408_ = 1.144, *p* = 0.309; treatment: F_1,408_ = 0.012, *p* = 0.912; time of the day: F_17,408_ = 9.289, *p* < 0.001).

### 2.4. Effects of the Selective MT_3_ Agonist GR135531 and Prazosin Injected at the End of the Light Phase on T_b_

**Figure 5:**
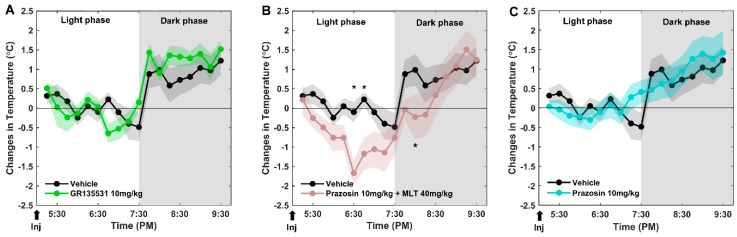
(**A**) Changes in T_b_ after the administration of the MLT MT_3_ agonist GR135531 (10 mg/kg) during the dark phase. (**B**) Prazosin (10 mg/kg) pre-treatment does not block the effects of MLT on T_b_ during the light phase. (**C**) Prazosin (10 mg/kg) injected during the light phase does not affect T_b_. Data are expressed as mean ± SEM (graded shades). Lights off at 7:30 p.m. * *p* < 0.05 versus vehicle; two-way ANOVA for repeated measures followed by Bonferroni *post hoc* test. Inj: s.c. injection of either vehicle, GR135531, MLT + prazosin, or prazosin.

As indicated in Figure 5A, the injection of GR135531 (10 mg/kg) at the end of the light phase (5:00 p.m.) did not affect T_b_ during the end of the light phase, the dark–light transition or the beginning of dark phase (two-way repeated measures ANOVA; interaction: F_17,306_ = 0.94, *p* = 0.527; treatment: F_1,306_ = 0.342, *p* = 0.566; time of the day: F_17,306_ = 7.817, *p* < 0.001). The effects of MLT on T_b,_ when injected during the light phase, were not mediated by MT_3_ receptors, since the pre-treatment with the non-selective α_1_/MT_3_ antagonist prazosin at a dose not affecting T_b_ (Figure 5C, interaction: F_17,357_ = 1.01, *p* = 0.446; treatment: F_1,357_ = 0.022, *p* = 0.882; time of the day: F_17,357_ = 7.588, *p* < 0.001) did not block the effects of MLT (Figure 5B; interaction: F_17,340_ = 1.65, *p* = 0.050; treatment: F_1,340_ = 3.064, *p* = 0.095; time of the day: F_17,340_ = 10.086, *p* < 0.001). Interestingly, the treatment with prazosin plus MLT induced a further decrease of T_b_ even during the dark phase at 8:00 p.m. (Figure 5B) that was not observed with MLT (Figure 2B) or prazosin (Figure 5C) alone.

## 3. Discussion

In this study, we investigated the effects of MLT and its three receptors on T_b_ during the light and the dark phase for the first time. To achieve this aim, we used a pharmacological approach employing MLT, the selective MT_1_ receptor partial agonist UCM871, the selective MT_2_ receptor partial agonist UCM924, the MT_3_ receptor agonist GR135531, and selective/non-selective MLT receptor antagonists, including the MT_2_ selective antagonist 4P-PDOT, the MT_1_/MT_2_ non-selective antagonist luzindole, and the MT_3_/α_1_ antagonist prazosin. The exogenous administration of MLT during the light phase decreased T_b_ immediately after the administration and before the light–dark phase shift, an effect blocked by both 4P-PDOT and luzindole. Interestingly, unlike MLT, neither UCM924 nor UCM871 produced a change in T_b_ during the light phase. In contrast, the selective MT_2_ partial agonist UCM924 administered at the end of the dark phase decreased T_b_ during the dark phase, just prior to the dark–light switch, whereas the selective MT_1_ partial agonist UCM871 injected at the end of the light phase increased T_b_ during the following dark phase. On the other hand, MT_3_ receptors did not seem to be involved in the regulation of T_b_, since the MT_3_ receptor agonist GR135531 and the MT_3_/α_1_ antagonist prazosin alone had not produced any effect on T_b_.

The rat circadian body temperature displays a cosine wave [39], showing a temperature that oscillates around the 35.6–36 °C during the light phase and around 37.8–38 °C during the dark phase [43]. The daily change in T_b_ shows a characteristic deviation at two different times, consistent with the switch between day (light phase) and night (dark phase) hours [44]. The present study replicates the same physiological T_b_ deviation that was previously reported in nocturnal rodents [43,44], with a daily T_b_ peak during the night, which is concomitant with the increase in the activity of the animals [45].

In nocturnal rodents, T_b_ peaks during night time when MLT levels are high, and decreases during the light phase when MLT levels are low. In contrast, in diurnal species, T_b_ regulation follows the reverse direction in relation to MLT levels, showing a T_b_ peak during the light phase [45] when circulating levels of MLT are very low (~10 pg/mL) [12,15,46]. However, the mechanism by which MLT regulates T_b_ has not been established.

The neuronal circuit controlling the regulation of T_b_ involves several structures including the SCN, SON, mPOA, and DMH [4,5]. Notably, the hypothalamus is an area rich in MLT receptors [11], and their expression may vary according to the phase and the time of the day [47,48,49,50,51].

Previous reports have shown that exogenous MLT administration during the light phase induced a decrease in T_b_ in both humans and rodents [32,35,36,37,39,52], although the active doses in humans are lower than those in rodents due to a significantly faster metabolism and very short half-life of MLT in the latter [53]. Our findings confirm that exogenous MLT influences T_b_, and its effects are strictly dependent on the time of day: MLT reduces T_b_ only towards the end of the light phase and if administered during the light phase. In regard to its possible mechanism of action during the light phase, we found that both the selective MT_2_ antagonist 4P-PDOT and the non-selective MT_1_/MT_2_ antagonist luzindole blocked the T_b_ reduction due to MLT, yet neither the selective MT_1_ partial agonist UCM871 nor the selective MT_2_ partial agonist UCM924 recapitulated the effects of MLT on T_b_. These findings suggest that during the light phase, MLT needs to simultaneously activate both MT_1_ and MT_2_ receptors to modulate T_b_. Indeed, the selective activation/inhibition of only MT_1_ or MT_2_ receptors did not affect T_b_ during the day. In contrast, UCM871 and UCM924 produced changes in T_b_ at different times of the dark phase and of opposite magnitude: UCM871 enhanced T_b_ just after the light–dark transition, whereas UCM924 decreased T_b_ just before the dark–light transition. Importantly, unlike UCM871 and UCM924, MLT did not induce any change in T_b_ during the dark phase. We previously observed a similar time-of-day-dependent effect of MLT on sleep [23]. However, it is interesting that when α_1_ receptors/MT_3_ receptors were blocked by prazosin, MLT decreased T_b_ also during the dark phase. These complex findings observed during the dark phase are likely dependent on the fact that during the dark phase there is a significant increase in the endogenous levels of MLT, and thus the expression of the two MLT receptors [19], as well as the involvement of other receptors implicated in thermoregulation, such as α_1_ adrenoceptors [54], probably vary.

Interestingly, it is now well recognized that MLT receptors can form MT_1_/MT_2_ hetero-oligomers and also heteromers with other receptors, and from a functional point of view, their properties are different from those of the corresponding homomers [55,56]. Since MLT receptors as well as other receptors including α_1_-adrenoceptors are highly expressed in brain regions/nuclei involved in thermoregulation, we cannot exclude that some of the effects of MLT on T_b_ described here were mediated by these oligomers/heteromers. Future studies are needed to investigate the possible circadian variability in the formation and role of oligomers and/or heteromers of MLT receptors in hypothalamic nuclei regulating T_b_. Similarly, the potential contribution of nuclear receptors, among which RORs that are also activated by MLT [31], is worth investigating.

The phase-dependent response of T_b_ to exogenous MLT may depend not only on the changes in the density of MLT receptors across the light–dark cycle, but also on the relative distribution and function of MT_1_ and MT_2_ receptors that control unique physiological responses in the brain, for example in sleep [21,22,23], anxiety [18,24], pain [25,26,57], and depression [28,58,59], and in the periphery, for example at the cardiovascular level [60,61].

In conclusion, we have investigated the role of MLT receptors in thermoregulation, and found that during the light phase T_b_ is affected only if both MT_1_ and MT_2_ receptors are simultaneously activated. Further, during the dark phase, a time-dependent effect was found in that the activation of MT_1_ and MT_2_ produces an increase and decrease of T_b_ respectively. No effects on T_b_ of the MLT MT_3_ receptor subtype were evidenced. However, MT_1_ and MT_2_ receptors control T_b_ in synergy with other receptors including α_1_ adrenoceptors. These data further support the recent findings showing that the MT_1_ and MT_2_ receptors modulate physio-pathological functions in different and sometimes opposing ways, and in a time-of-day dependent manner. In particular, MT_1_ or MT_2_ agonists may be further tested for hypothermia or hyperthermia, respectively.

## 4. Materials and Methods

### 4.1. Animals

Male Wistar rats (200–250 g, Charles River) were used for behavioral tests. All animals were housed at constant room temperature (20 °C) and humidity under a 12/12-h light–dark cycle (lights on at 7:30 a.m. and off at 7:30 p.m.) with food and water ad libitum. All experimental procedures were performed between 5:00 a.m. and 9:30 a.m. and between 5:00 p.m. to 9:30 p.m. The experimental protocol was approved by the Animal Ethics Committee (AUP#5253, McGill University, QC, Canada) and followed the ethical guidelines of the Canadian Institute of Health Research for animal care and scientific use.

### 4.2. Drugs and Pharmacological Treatments

**Figure 6:**
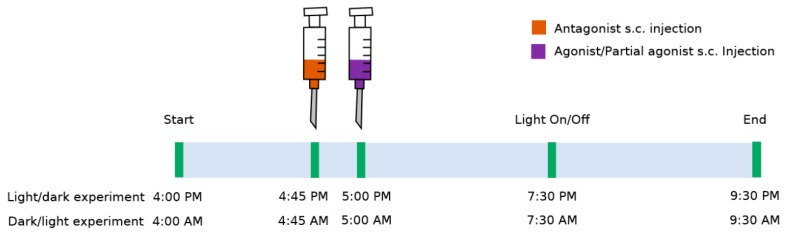
Schematic representation of the experimental design. Body temperature (T_b_) has been recorded every 15 min. For light–dark phase experiments, light was off at 7:30 p.m., whereas for dark–light phase experiments, light was on at 7:30 a.m. s.c.: subcutaneous.

The N-(2-{Methyl-[3-(4-phenylbutoxy)phenyl]amino}ethyl)acetamide (UCM871, 14 mg/kg) [42] and N-{2-[(3-bromophenyl)-(4-fluorophenyl)amino] ethyl}acetamide (UCM924, 40 mg/kg) [41] were synthesized by the University of Urbino, Italy, and by BioQuadrant Inc. (Montreal, Canada), respectively. Melatonin (40 mg/kg), luzindole (10 mg/kg), and prazosin hydrochloride (1-(4-Amino-6,7-dimethoxy-2-quinazolinyl)-4-(2-furanylcarbonyl)piperazine hydrochloride; 10 mg/kg) were purchased from Sigma (St. Louis, MO, USA), and 4P-PDOT (4-phenyl-2-propionamidotetralin; 10 mg/kg) and GR135531 (5-Methoxycarbonylamino-N-acetyltryptamine, 10 mg/kg) from Tocris Bioscience (Ellisville, MO, USA). All drugs were dissolved in a vehicle composed of 70% dimethyl sulfoxide (MP Biochemicals, Solon, OH, USA) and 30% saline. The doses of UCM924, MLT, 4P-PDOT, and luzindole [23,25,26], as well as UCM871 [62], were chosen based on our previous experiments examining the potential pharmacological activity of these compounds. The doses of GR135531 and prazosin were based on the literature [63,64]. Drugs were injected subcutaneously (s.c.; 0.5 mL) 15 min before the beginning of the experiment: 5:00 a.m. for dark–light or 5:00 p.m. for light–dark testing. The selective MLT MT_2_ receptor antagonist 4P-PDOT, the non-selective MLT MT_1_/MT_2_ receptor antagonist luzindole, and the MT_3_/α_1_ antagonist prazosin (pKi = 21.7) [65] were injected 15 min prior the agonist/partial agonist. Figure 6 describes the experimental protocol.

### 4.3. Assessment of Body Temperature

Body (rectal) temperature (T_b_) in awake animals was measured by goosing the animal using a Traceable Snap-in Module with probe (Fisher Science Education, S90862). The probe was inserted to a depth of 2 cm for no more than 10 s, whereas the tested individual was kept in a cotton bag. Animals were handled every day for five days before the experiments with the aim of habituating the animal to the testing procedure and thus minimizing the associated stress.

### 4.4. Statistical Analysis

Data analysis was conducted using the SigmaPlot statistical software version 13 (Systat Software, Inc.). After controlling for the normal distribution of the data, a two-way ANOVA for repeated measures was used to analyze the data using treatments (between) and testing time (within) as factors. Post hoc analyses were performed using the Bonferroni test for multiple comparisons. The effect of vehicle was compared with that of the different agonists/partial agonists, the antagonists alone, or the agonist/partial agonist plus the antagonist. All data were expressed as mean ± SEM. *p* < 0.05 was considered significant. All figures were made using MATLAB software.

Temperature values were normalized as follow: [Temperature at time X – Average of Temperature (4:00 a.m./p.m. to 5:00 a.m./p.m.)]/[Average of Temperature (4:00 a.m./p.m. to 5:00 a.m./p.m.)]. “Time X” indicates any time after the injection.
